# Biosorption of Uranium from aqueous solution by green microalga *Chlorella sorokiniana*

**DOI:** 10.1007/s11356-022-19827-2

**Published:** 2022-04-02

**Authors:** Mohamed A. Embaby, El-sayed A. Haggag, Ahemd S. El-Sheikh, Diaa A. Marrez

**Affiliations:** 1 Food Toxicology and Contaminants Department, National Research Centre, Cairo, Egypt; 2 Nuclear Materials Authority, El-Kattamia, El-Maadi, Cairo, Egypt

**Keywords:** Biosorption, Uranium, Green microalga, *Chlorella sorokiniana*

## Abstract

Uranium and its compounds are radioactive and toxic, as well as highly polluting and damaging the environment. Novel uranium adsorbents with high biosorption capacity that are both eco-friendly and cost-effective are continuously being researched. The non-living biomass of the fresh water green microalga *Chlorella sorokiniana* was used to study the biosorption of uranium from aqueous solution. The biosorption of uranium from aqueous solutions onto the biomass of microalga *C. sorokiniana* was investigated in batch studies. The results showed that the optimal pH for uranium biosorption onto *C. sorokiniana* was 2.5. Uranium biosorption occurred quickly, with an equilibrium time of 90 min. The kinetics followed a pseudo-second-order rate equation, and the biosorption process fit the Langmuir isotherm model well, with a maximum monolayer adsorption capacity of 188.7 mg/g. The linear plot of the DKR model revealed that the mean free energy *E* = 14.8 kJ/mol, confirming chemisorption adsorption with ion exchange mode. The morphology of the algal biomass was investigated using a scanning electron microscope and energy dispersive X-ray spectroscopy. The FTIR spectroscopy analysis demonstrated that functional groups (carboxyl, amino, and hydroxyl) on the algal surface could contribute to the uranium biosorption process, which involves ion exchange and uranium absorption, and coordination mechanisms. Thermodynamic simulations indicated that the uranium biosorption process was exothermic (ΔH = −19.5562 kJ/mol) and spontaneous at lower temperatures. The current study revealed that *C. sorokiniana* non-living biomass could be an efficient, rapid, low-cost, and convenient method of removing uranium from aqueous solution.

## Introduction

Uranium is a naturally occurring radioactive element with a specific density of 19 g/cm^3^ that can be found in the Earth’s crust in many chemical and physical forms. Uranium can be found in nature in complex ores such as uraninite, pitchblende, autunite, carnotite, torbernite, and uranophane. Pitchblende uraninite is the principal mineral of uranium, accounting for around 50 to 80% of total ore (Francis 1994; Choppin et al. 2013). Uranium is found in the Earth’s crust at a concentration of around 2.8 mg/kg. However, uranium released into the environment is a result of several anthropogenic activities such as nuclear weapon testing, mineral mining, and the use of natural leaching of uranium-bearing rocks and uranium-containing phosphate fertilizers (Anke et al. 2009; Todorov 2004). Uranium and its compounds are radioactive and toxic, as well as very polluting and damaging to the environment. It can eventually make its way into drinking water and food and be consumed by humans, causing serious liver, kidney, and bone illnesses (Yi et al. 2017a, 2017b; Brugge and Buchner 2011; Thiebault et al. 2007; Katsoyiannis et al. 2007; Kurttio et al. 2005 and Domingo 2001). Uranium bioaccumulation in the food chain may render higher consumers, particularly humans, more vulnerable to the negative effects of uranium exposure (Carvalho et al. 2014). When consuming uranium at levels above the allowable limit, there is a considerable danger of physical distortion and a variety of diseases (Soltani et al. 2019; ATSDR 2011). When uranium enters the bloodstream, it forms a variety of complexes, including uranyl bis- and tris-carbonate complexes, as well as UO2-protein complexes with human serum albumin, transferrin, and other proteins. Uranium has a strong affinity for phosphate groups and can attach to phosphorylated peptides (Pardoux et al., 2012). Uranium has been linked to nephrotoxicity, genotoxicity, and bone carcinogenicity (Brugge and Buchner 2011). Toxicological data has been acquired via sub-acute, acute, sub-chronic, and chronic exposure of different organisms to uranium. All uranium species have been shown to have effects on the kidney, specifically proximal tubule damage, and glomerulus damage at large dosages. Even if injured cells repair if the exposure is insufficient, minor morphological changes with inexact effects have been seen (Health Canada 1987; COT 2006; EFSA 2009). Besides nephrotoxicity, probable evidence of uranium toxicities such as carcinogenicity, neurotoxicity, and reproductive toxicity has been found in animals and/or humans (Craft et al. 2004). As a result, less than 30 mg/L uranium has been suggested by WHO in health-based drinking water quality guidelines (WHO 2011). It has also been shown that the toxicity of uranium causes increased oxidative damage, membrane permeability, and transient RNA degradation (Kolhe et al. 2020). Human exposure occurs through the consumption of food, water, and inhalation. Inhalation exposure is considered minimal (0.0015 μg/day, excluding occupational exposure), and uranium consumption from water and food is equivalent to 0.9–1.5 μg/day (EFSA 2009; ATSDR 2009). The WHO estimates total uranium consumption by food of 1–4 μg/person/day, a value supported by research in Switzerland, which found an average intake of 3.7 μg/person/day (excluding drinks). High uranium levels in drinking water are a global problem. Ground water uranium contamination has been reported in various countries, including China (Wang et al. 2012), Brazil (Godoy et al. 2019), India (Pant et al. 2019), Pakistan (Ali et al. 2019), and Argentina (Matteoda et al. 2019). Drinking uranium-contaminated water is hazardous to human health (Pant et al. 2017). Shin et al. (2016) found high uranium level above 30g/L in 160 of 4140 groundwater wells in Korea, most of which were located in plutonic bedrock locations. The greatest observed quantities of uranium in foodstuffs variety from the USA and UK were found in shellfish, ranging between 9.5 and 31 μg/kg (EFSA 2009). According to Neves et al. (2012), high concentrations of uranium were detected in irrigation waters (218–1,035 μg/l), in some soils (U total > 50 mg/kg), and in some vegetable foodstuffs (1.6, 16, 22, 26, 30, 110, and 234 μg/kg fresh weight for apple, carrot, corn, cabbage, green bean pods, potato with peel, and lettuce respectively). Typical uranium level in staple foods like fresh vegetables and bread was found to be around 2 μg/kg, but uranium content in other foods like meat and rice was found to be in the range of 0.1 to 0.2 μg/kg (WHO 2001). As a result, uranium removal from polluted areas becomes critical for regulatory compliance guidelines and environmental safety (Alqadami et al. 2017; Acharya 2015). Adsorption (Abdi et al. 2017; Solgy et al. 2015), membrane filtration (Ding et al. 2016; Torkabad et al. 2017; Reguillon et al. 2008), ion exchange (Ladeira and Gonçalves 2007; Huikuri and Salonen 2000), and electrodialysis have all been used to eliminate uranium from groundwater (Onorato et al. 2017; Montana et al. 2013). Adsorption technology has been widely employed because of its high removal effectiveness and ease of operation and maintenance (Solgy et al. 2015; Abdi et al. 2017; Embaby et al. 2021). Various adsorbents have been examined for potential application, which include natural minerals like diatomite, hematite, clinoptilolite, montmorillonite (Jang et al. 2007; Sylwester et al. 2000; Sprynskyy et al. 2010; Camacho et al. 2010), and biomaterials such as *Cystoseria Indica* algae and *Bacillus subtilis* (Khani et al. 2008; Fowle et al. 2000) and synthesized materials such as modified silica, various iron-based materials, polymers, and carbon-based adsorbents (Lee et al. 2010; Fan et al. 2012; Wei et al. 2016; Zhang et al. 2016; Chen et al. 2017; Wu et al. 2018; Li et al. 2019). Novel adsorbents with high uranium adsorption capacity that are both eco-friendly and cost-effective are still being investigated (Lee et al. 2019). Therefore, the current study was carried out to investigate the usage of *Chlorella sorokiniana* algal biomass for uranium removal from aqueous solution by biosorption under various parameters such as contact time, pH, temperature, adsorbent dosage, and initial uranium ion concentration. The adsorption processes and thermodynamics were also examined.

## Materials and methods

### Chemicals and reagents

All chemicals used for analysis were analytical grade reagents. Uranyl sulfate trihydrate UO_2_SO_4_·3H_2_O from IBI labs, Florida, USA, and HCl 37%, HNO_3_, NaCl, CH_3_COONa, and H_2_SO_4_ 98% was obtained from Fisher, Arsenazo III from Sigma-Aldrich, USA.

### Preparation of algal biomass (microalgae strain, cultivation, and production)

The microalgae employed for the current study was *Chlorella sorokiniana* SAG 211-8k that was obtained from Marine Toxin laboratory, National Research Centre, Egypt (Faried et al. 2021). The culture medium used for cultivation was BG-11 (Rippka et al. 1979), and BG-11 medium is composed of 1.5 g NaNO_3_, 0.004 g K_2_HPO_4_, 0.075 g MgSO_4_·7H_2_O, 0.036 g CaCl_2_·2H_2_O, 0.006 g citric acid, 0.02 mg Na_2_CO_3_, 0.001 g Na_2_EDTA, 0.63 g ferric ammonium citrate, and 1.0 mL trace elements (TE) in 1000 mL distilled water. TE (g/l) is combined of 2.86 g H_3_BO_3_, 1.81 g MnCl_2_·4H_2_O, 0.222 g ZnSO_4_·7H_2_O, 0.39 g Na_2_MoO_4_·2H_2_O, 0.079 g CuSO_4_·5H_2_O, and 0.0494 g Co(NO_3_)_2_·6H_2_O. After autoclaving and cooling, pH was adjusted to 7.1. Laboratory production of *C. sorokiniana* was performed using glass flasks 5 L containing 3 L algal growth medium. The cultivation was done under continuous illumination provided from white fluorescent lamps at room temperature and aeration was performed using air compressor linked with polyethylene tubes (3 mm). After 25 days, at stationary phase of growth, *C. sorokiniana* biomass were harvested using centrifuge (SIGMA Laborzentrifugen Gmbh) at 4000 ×g for 15 min and dried in a hot air oven at 50°C for 2–4 h.

### Adsorbent characterization

The surface morphology of *C. sorokiniana* biomass before and after loading with the uranium ions was examined by scanning electron microscope (SEM) Model Philips XL 30 coupled with EDX (operating conditions: 25–30 kv accelerating voltage, 1–2 mm beam diameter, and 60–120 s counting time). The samples were gold-coated before observation to enhance the electrical conductivity. Functional groups on the surface of *C. sorokiniana* were observed using FTIR Bruker VERTEX 80 (Germany) combined Platinum Diamond ATR, which comprises a diamond disk as that of an internal reflector in the range 4000–400 cm^−1^ with resolution 4 cm^−1^, refractive index 2.4.

### Determination of point of zero charge (pH_PZC_)

The pH_PZC_ of the *C. sorokiniana* biomass was determined by using degassed 0.01 M NaCl solution, at 298 K. In different 100-mL conical flasks, 50 mL of the 0.01 M NaCl solution was added and the pH was adjusted at 2, 4, 6, 8, 10, 12, and 14 using 0.5 M HCl or 0.5 M NaOH. Then 50 mg of *C. sorokiniana* biomass was added to each of the above pH adjusted solutions and equilibrated for 24 h. The final pH values of the solutions were recorded and the difference between the initial and final pH (the so-called Δ pH) was plotted against the initial pH values. The PZC values were calculated from Δ pH vs initial pH plot, at the pH where Δ pH = 0 (Nasiruddin and Sarwar 2007).

### Batch experiments for uranium adsorption

In our preliminary tests, the uranium adsorption efficiency was very high with small dosages of *Chlorella sorokiniana* biomass (0.02 g). The adsorption of uranium on algal biomass was examined by batch technique and the effects of various parameters on the rate of adsorption process were observed by varying pH of the solution, contact time, temperature, adsorbent concentration, and initial ion concentration. For adsorption studies, known amount of powdered algal biomass was shaking with 10 mL uranyl sulfate solution (concentration range of uranium: 400–3000 mg/L) at various pH (0.2–4) at different temperatures (25–65 C°) in 50 mL well-sealed polypropylene bottles. Contact time and adsorbent doses were altered from 5 to 120 min and 0.008–0.1 g respectively. Before adding algal biomass, the pH of the solutions was adjusted by adding a negligible volume of 0.01 or 0.1 mol/L HCl and/or NaOH solutions. Throughout the adsorption experiments, 250 rpm was the speed maintained for shaking. The adsorption efficiency (percent metal ion removal) was calculated according to relation (1):

1 $${\mathrm{{Uranium}\;{biosorption}\;{efficiency}}}\;\%=\frac{C_o-C_e}{C_o}\times100$$

where *C*_o_ and *C*_e_ are the initial and equilibrium uranium concentrations in aqueous solution (mg/L), respectively. The adsorption capacity at equilibrium (*q*_e_, mg/g), i.e., quantity of uranium adsorbed by unit mass of algal biomass, was determined by following relation (2):

2 $${q}_e=\left({C}_o-{C}_e\right)\times \frac{V}{m}$$

where *V* is the volume of solution (L), and *m* is the weight of the biomass (g).

The distribution coefficient (*K*_d_) of uranium between the aqueous bulk phase and the solid phase was calculated from the following relation (3):

3 $${K}_d=\frac{C_o-{C}_e}{C_e}\times \frac{V}{m}$$

### Kinetic modeling

Kinetic models are usually employed to describe the rate-determining step of the adsorption process. Two commonly used kinetic models, namely, pseudo-first-order and pseudo-second-order, were selected to analyze the kinetic data and to understand the rate-determining step of uranium adsorption onto *C. sorokiniana* biomass. The pseudo-first-order equation is a simple kinetic model describing the kinetic process of liquid-solid phase sorption and its linear formula can be written as follows:

4 $$\log \left({q}_e-{q}_t\right)=\log {q}_e-\left[\frac{k_1}{2.303}\right]t$$

where *k*_1_ is the rate constant of the pseudo-first-order sorption (min^−1^). Evidently, *k*_1_ can be calculated from the slope of the plot of log (*q*_*e*_*−q*_*t*_) versus *t*. The pseudo-second-order model based on the adsorption equilibrium capacity may be expressed in the following linear form:

5 $$\frac{t}{q_t}=\frac{1}{k_2{q}_e^2}+\left[\frac{1}{q_e}\right]t$$

where *k*_2_ is the rate constant of pseudo-second-order adsorption (g/(mg·min)). Evidently, *q*_e_ and *k*_2_ can be determined experimentally by plotting *t*/*q*_*t*_ versus *t*.

### Equilibrium modeling

Three adsorption isotherm models, namely, Langmuir Freundlich and Dubinin-Kaganer-Radushkevich (DKR) were selected to correlate the experimental data and to describe the adsorption isotherms exactly. The deviation between experimentally observed and theoretically calculated data can be described by the square of the correlation coefficient (*R*2). The linear Langmuir equation can be written as follows:

6 $${{c}_e}\left/{{q}_e}\right.= {1}\left/ {b.{q}_{\mathrm{max}}}\right.+{{c}_e}\left/ {{q}_{\mathrm{max}}}\right.$$

where *q*_max_ is the maximum possible amount of metals adsorbed per unit of weight of adsorbent (mg/g) and *b* is a constant associated with the affinity of binding sites for metals (L/mg). *q*_max_ and *b* can be determined from the plot of *C*_e_/*q*_e_ versus *C*_e_. The Freundlich isotherm may be suitable for nonideal uptake onto heterogeneous surfaces involving multilayer adsorption (Freundlich, 1906). The linear Freundlich equation can be expressed as follows:

7 $$\log {q}_e=\log {K}_f+{1}\left/{n}\right.\log {C}_e$$

where *K*_*f*_ is the Freundlich constant representing the adsorption capacity of the adsorbent and *n* is the Freundlich exponent representing adsorption intensity. *K*_*f*_ and *n* can be determined from the plot of log *q*_e_ versus log *C*_e_.

The equilibrium data were also fitted by the DKR model to distinguish between physical and chemical adsorption (Dubinin and Serpinsky 1981; Boparai et al. 2011). The linear form of the DKR isotherm is expressed as follows:

8 $$\ln {q}_e=\ln {X}_m-{\beta \upvarepsilon}^2$$

where *q*_e_ is adsorption capacity at equilibrium (mol/g), *X*_*m*_ is the theoretical DKR monolayer adsorption capacity (mol/g), *β* (mol^2^/J^2^) is a constant associated with adsorption energy, and *ε* (J/mol) is the Polanyi potential related to the equilibrium concentration and can be defined as follows:

9 $$\upvarepsilon = RT\ \mathit{\ln}\left(1+{1}\left/ {{C}_e}\right.\right)$$

The mean adsorption energy, *E* (kJ/mol), can be derived by using the following equation (Wang et al. 2015):

10 $$E={1}\left/{\sqrt{2\beta }}\right.$$

### Analytical method

The concentration of uranium (VI) in the aqueous solution (*C*_eq_, mg/L) was analyzed spectrophotometrically at 650 nm using Arsenazo III dye (Haggag 2021) (Shimadzu UV–VIS-1601 spectrophotometer).

### Elution studies

Elution of uranium from the loaded algal biomass was studied using various eluting mediums such as HCl, H_2_SO_4_, Na_2_CO_3_, HNO_3_, NaOH, Na_2_SO_4_, CH_3_COONa, and NaCl. The elution experiments were conducted by shaking 0.1 g of the loaded adsorbent with 10 mL of different eluates each separately for 60 min at 250 rpm. After filtration, the eluting solution was analyzed against uranium (El-Sheikh et al. 2020).

## Results and discussion

### Adsorbent characterization (surface morphology of C. sorokiniana biomass)

**Fig. 1:**
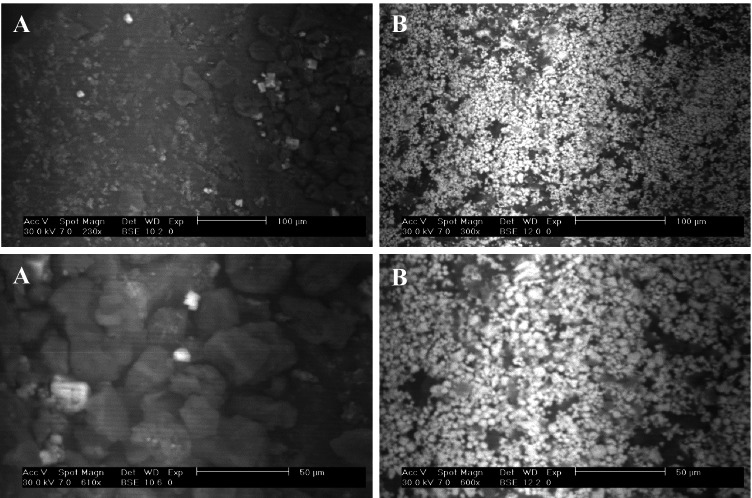
SEM images of *C. sorokiniana* biomass before (**A**) and after (**B**) uranium biosorption process

The SEM images of *C. sorokiniana* biomass before and after adsorption of uranium indicate a variation in biomass morphology as shown in Fig. 1. The images before adsorption show a rough surface algal biomass, with many voids. However, SEM images after adsorption show decrease of the voids and appearance of bright areas in the biomass surface; this indicates a significant change in the surface morphology of algal biomass. This significant change could be attributed to the presence of uranium that adsorbed by algal biomass in adsorption process. This may be due to the interaction of uranium with functional organic groups (e.g., the carboxylic and hydroxyl groups) of the cell walls biomass, the exchange of H^+^ on the surface with uranium, and diffusion of free uranium into the void of the algal biomass (Mahmoud et al. 2015; Khawassek et al. 2017; Khawassek et al. 2019; Abdel-Samad et al. 2020; Dacrory et al. 2020).

### Optimization of uranium adsorption conditions

#### Point of zero charge (pH_PZC_)

**Fig. 2:**
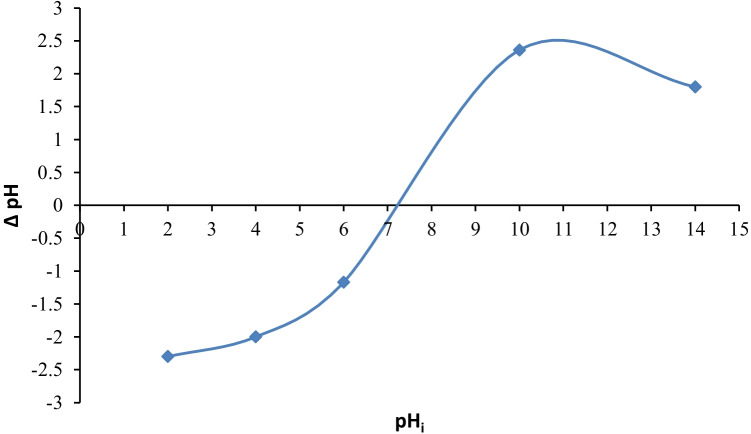
pH_PZC_ determination curve of *C. sorokiniana* biomass

The point of zero charge is abbreviated as pH_PZC_, the pH at which the adsorbent’s net surface charge is equal to 0 (Saleh 2015). In the current study, the pH_PZC_ of *C. sorokiniana* biomass was calculated using the pH drift method (Chutia et al. 2009). Figure 2 represents a graph of Δ pH vs initial pH, and the pH_ZPC_ *C. sorokiniana* biomass was determined to be 7.2.

#### Effect of pH

**Fig. 3:**
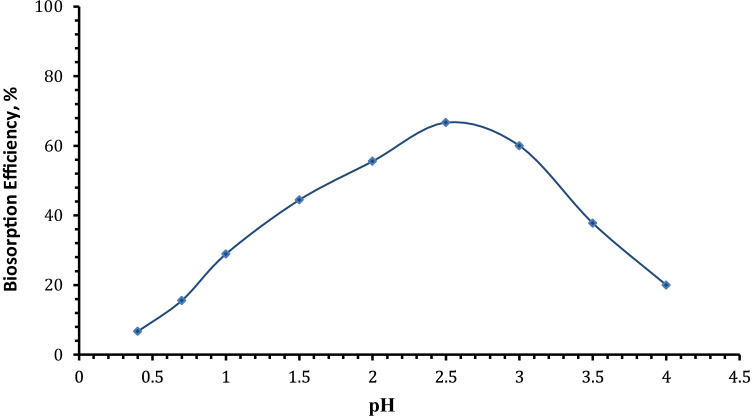
Effect of pH on uranium uptake by *C. sorokiniana* biomass (*v*: 10 mL, *C*_0_: 450 mg/L, *w*: 0.02 g algal biomass, *T*: 25°C, rpm: 250, contact time: 120 min)

Uranium biosorption is strongly dependent on pH value of the solution because both surface charge of the adsorbent and speciation change as a function of pH (Razdan and Shoesmith 2014). The effect of pH on the uranium ion biosorption efficiency from aqueous solution by *C. sorokiniana* biomass was investigated at pH values ranging from 0.3 to 4 (as it was discovered that increasing the pH value above 2.75 cause partial precipitation of uranium ions) while the other parameters were held constant using 0.02 g algal biomass, and 10 mL aqueous solution assaying 450 mg U/L for 120-min contact time at room temperature. It is clear from the results of Fig. 3 that with increasing the pH, the adsorption efficiency of *C. sorokiniana* biomass increased gradually till reaching the maximum value of 66.6% at pH value 2.5.

The low adsorption capacity at lower pH is due to the high concentrations of H^+^ in the reaction mixture, increasing positivity of adsorbents and electrostatic repulsion between positively charged uranyl cations (Ai et al. 2013; Cao et al. 2013). The biosorption of positively charge uranium on the cell surface of algal biomass was enhanced by increasing pH, where increasing pH led to an increase of the negative charges on the algal biomass due to deprotonation. The biosorption of uranium decreased gradually after the pH value of 2.5. Therefore, pH = 2.5 was selected as the optimum pH for the subsequent biosorption experiments.

**Fig. 4:**
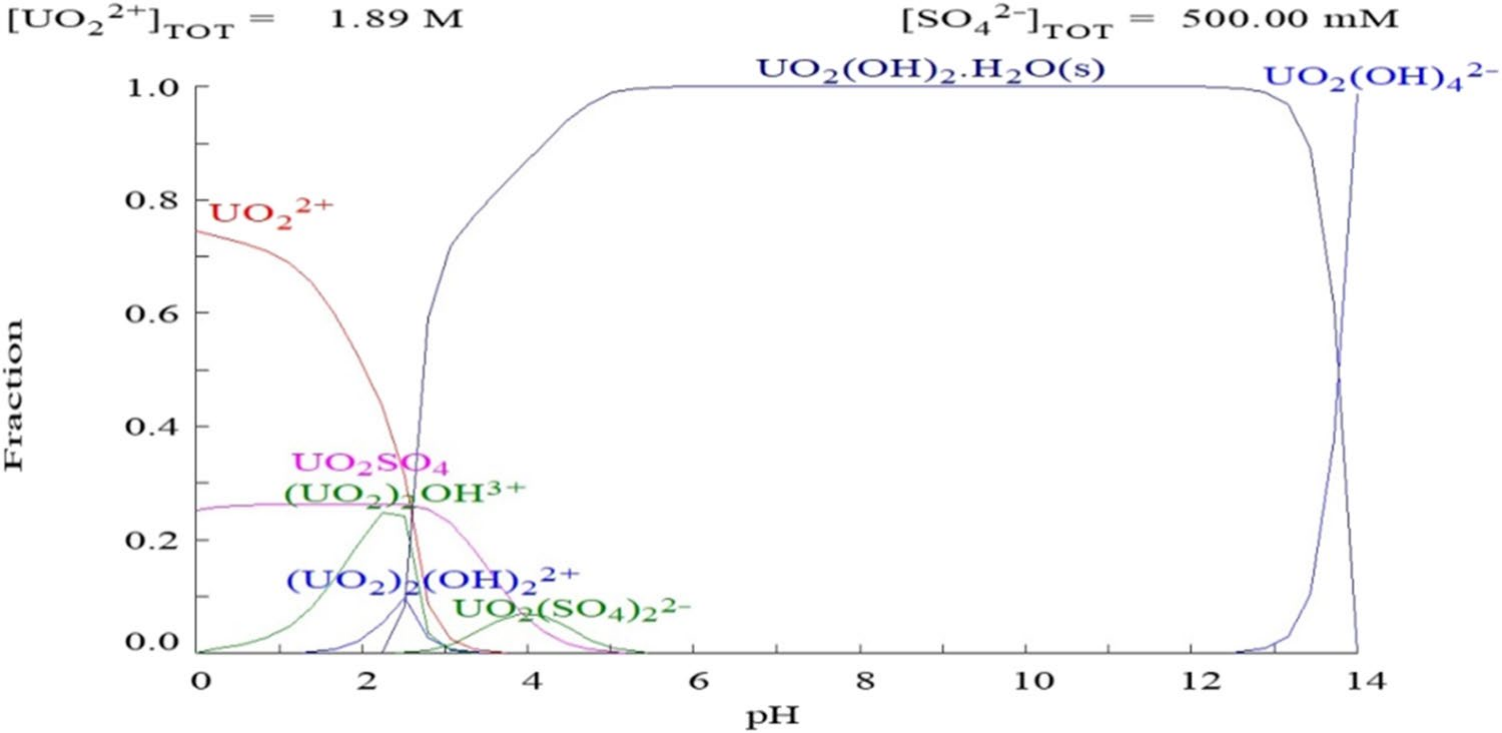
Expected aqueous speciation of uranium (600 mg/L) as a function of pH in 0.5 M H_2_SO_4_ using Medusa and Hydra program

The aqueous speciation distribution of uranium is represented in Fig. 4. The results observed that the complexes of UO_2_SO_4_ and UO_2_ (SO_4_)_2_^2−^ were the predominant species at pH range from 0.0 to 5.5 with a mean total percent of 8.33 and 75%, respectively, at pH 0 while 18 and 82% at pH 5.5. U-hydroxide complexes start to dominate the aqueous phase at pH near 6. The dominate complex of UO_2_ (OH)_2_·H_2_O became the major species with about 100% of total concentration at pH range from 6 to 12. After pH 12, UO_2_ (OH)_4_^2−^ and UO_2_OH_3_^−^ became the major species (Haggag et al. 2010).

#### Effect of contact time

**Fig. 5:**
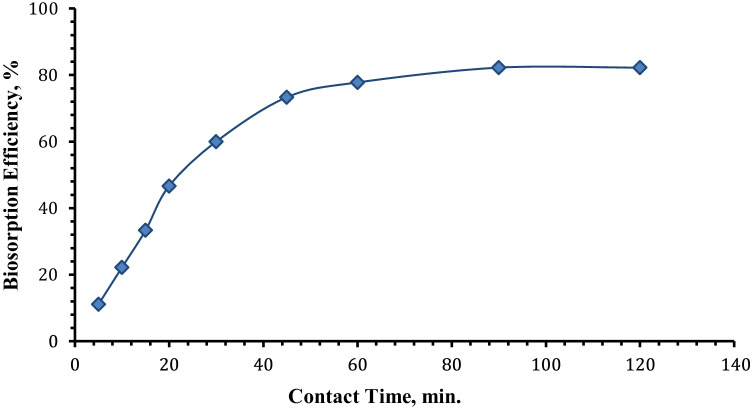
Effect of contact time on uranium biosorption efficiency using *C. sorokiniana* biomass (pH: 2.5, v: 10 mL, *C*_0_: 450 mg/L, *w*: 0.02 g biomass, *T*: 25°C, rpm: 250)

The influence of contact time on uranium biosorption efficiency utilizing algal biomass from 10 mL of aqueous solution assaying 450 mg U/L was tested from 4 to 120 min, while the other parameters were set at pH 2.5 and 0.02 g from algal biomass at room temperature. The plotted findings in Fig. 5 show that the uranium biosorption efficiency gradually rose with increasing contact time, reaching a maximum of 82.2 mg/g at 90 min and remaining constant thereafter. As a result, the adsorption equilibrium time chosen for further study is 90 min. The plots revealed that the kinetics of uranium biosorption consisted of two phases: the early rapid period where adsorption was fast and contributed greatly to equilibrium uptake, and the slower second phase where biosorption was very slow and contributed relatively little to total metal adsorption. The first phase is caused by immediate adsorption or external surface adsorption, whereas the second is caused by progressive adsorption, in which intraparticle diffusion limits the adsorption rate until metal uptake achieves equilibrium (Han et al. 2007).

#### Effect of adsorbent dose

**Fig. 6:**
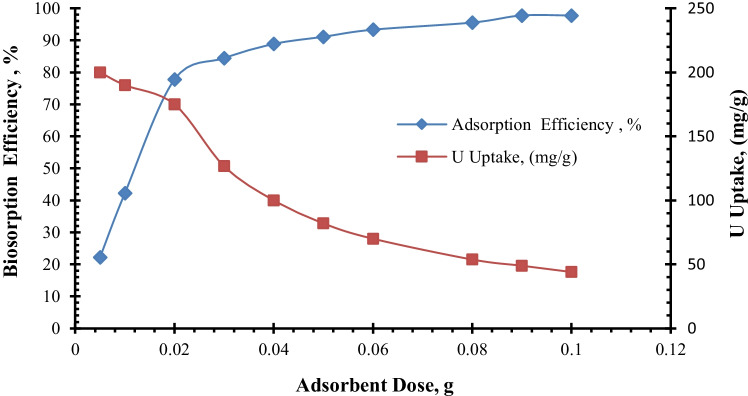
Uranium biosorption efficiency and uptake as a function of *C. sorokiniana* biomass dose (pH: 2.5, *v*: 10 mL, *C*_0_: 450 mg/L, contact time: 90 min, *T*: 25°C, rpm: 250)

Influence of the adsorbent dosage on uranium biosorption efficiency and uptake was studied for 90 min at room temperature with a uranium initial concentration of 450 ppm and a pH of 2.5. As presented in Fig. 6, the biosorption efficiency of uranium bioremoval from the aqueous solution was observed to increase concomitantly by increasing the dose of biomass, and the biosorption efficiency increases rapidly by the adsorbent dose increasing from 0.005 to 0.02 g, whereas it increases slowly from 0.03 to 0.06 g. Due to the low metal content in solution, increasing the biomass concentration from 0.06 to 0.1 g did not result in considerable elimination (Fourest and Roux 1992). At an adsorbent dosage of 0.09 g, the maximum biosorption efficiency of uranium removal was 97.7%. In contrast, as the adsorbent dosage was increased, the uranium biosorption capacity (mg/g) dropped. Various researchers hypothesize that high biomass concentration increases electrostatic interactions between cells, resulting in limited availability of binding sites (Deng et al. 2011; Li et al. 2016; Yuan et al. 2020), and reduces the total surface area for uranium ion biosorption due to overlapping and aggregation of the biomass cells (Alene et al. 2020; Zhang et al. 2019).

The decrease in uptake value of uranium with increasing biomass concentration is most likely due to a decrease in metal concentration in solution, where in the presence of high biomass concentration there is very fast superficial adsorption on to microbial cells, resulting in a lower concentration of metal in solution. Similar findings on the impact of biomass content on metal biosorption have been reported for a variety of microorganisms (Akhtar et al. 2007; Bayramoglu et al. 2018).

#### Effect of initial uranium concentration

**Fig. 7:**
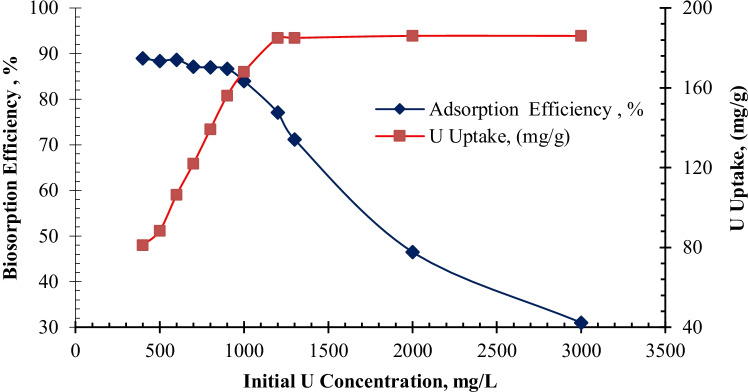
Effects of initial uranium ion concentration on biosorption efficiency and uptake by *C. sorokiniana* biomass (pH: 2.5, *v*: 10 mL, contact time: 90 min, *w*: 0.09 g biomass, *T*: 25°C, rpm: 250)

Effect of the initial uranium ion concentration on biosorption was examined by incubating 0.09 g of biomass for 90 min with 10 mL of uranium solutions varying in concentration from 400 to 3000 mg/l at a pH of 2.5. According to Fig. 7, the amount of uranium taken up by the biomass increased rapidly with increasing uranium concentration from 400–1200 mg/l. This increase could be attributed to an increased likelihood of collision between metal ions and biosorbent particles (Fourest and Roux 1992). A plateau was maintained with maximum uranium uptake of 186 mg/g from 1200 to 3000 mg/l, which could be interpreted as an important driving force to overcome the mass transfer resistance of uranium between the aqueous and solid phases (Yi et al. 2017a, 2017b). On the other hand, increasing the initial uranium concentration reduced the biosorption efficiency of uranium on algal biomass. This decrease can be interpreted that number of active sites on the biomass is decreased due to affinity of uranium ions to bind with the active sites as a result of the increase of the initial uranium concentration (Haggag et al. 2020).

#### Effect of temperature

**Fig. 8:**
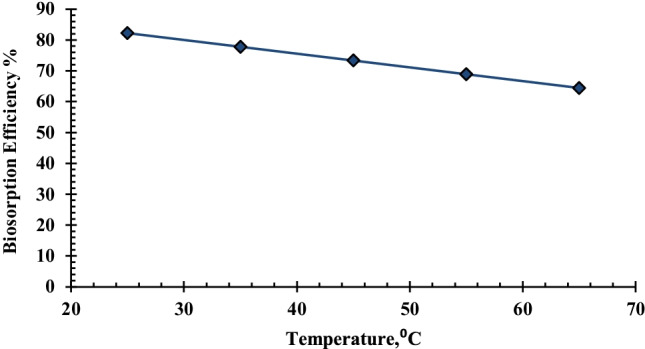
Effect of temperature on uranium biosorption efficiency and uptake by *C. sorokiniana* biomass (pH: 2.5, *v*: 10 mL, contact time: 90 min, *v*: 0.09 g biomass, *C*_0_: 450 mg/L, rpm: 250)

The influence of temperature on uranium biosorption was investigated at temperatures ranging from 25 to 65°C using 0.02 g biomass, a 10-mL solution of uranium with an initial concentration of 450 mg/L, and a constant pH of 2.5 over a 90-min contact time. According to the results shown in Fig. 8, the best biosorption temperature for the removal of uranium in aqueous solution using *C. sorokiniana* biomass is 25 °C. Increasing the temperature results in a decrease in removal efficiency, which can be attributed to deactivating the biosorbent surface or destroying some active sites on the biosorbent surface due to bond rupture (Meena et al. 2005) or due to the weakening of biosorption forces between the active sites on the surface of the *C. sorokiniana* biomass and the uranium ions; as temperature rises, the kinetic energy of adsorbed molecules rises, and they overcome the electrostatic force of attraction by the adsorbent surface (Vijayaraghavan and Yun 2008).

### Biosorption kinetics and mechanism

The adsorption kinetic characteristic was described using the pseudo-first-order model and the pseudo-second-order model. Equations 4 and 5 can be used to express the two models.

Pseudo-first order:

4 $$\log \left({q}_e-{q}_t\right)=\log {q}_e-\left[\frac{k_1}{2.303}\right]t$$

**Fig. 9:**
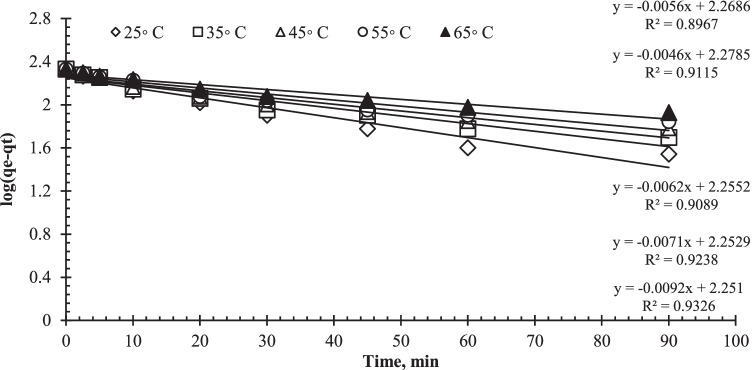
Lagergren plots for biosorption (pseudo-first order kinetics) of uranium on *C. sorokiniana* biomass (pH: 2.5, *V*: 10 mL, *C*_0_: 450 mg/L, *w*: 0.09 g algal biomass, rpm: 250)

**Fig. 10:**
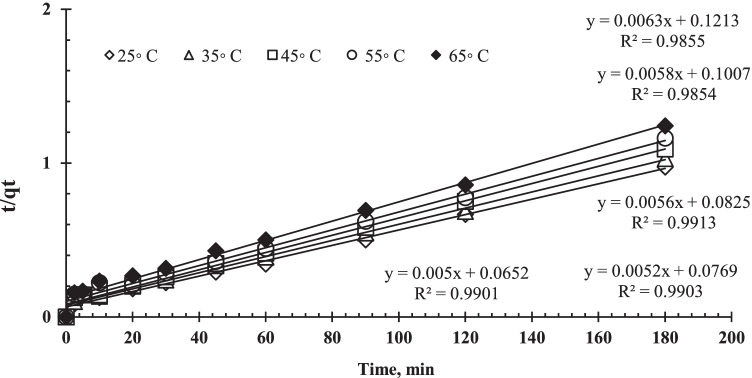
Pseudo-second order kinetics of uranium biosorption on *C. sorokiniana* biomass (pH: 2.5, *V*: 10 mL, *C*_0_: 450 mg/L, *w*: 0.09 g biomass, rpm: 250)

Pseudo-second-order:

5 $$\frac{t}{q_t}=\frac{1}{k_2{q}_e^2}+\left[\frac{1}{q_e}\right]t$$

where *q*_e_ is the uranium uptake quantity at equilibrium, *q*_*t*_ is the uranium biosorption capacity at any time (*t*), and *k*_1_ and *k*_2_ are the rate constants of pseudo-first- and pseudo-second-order sorption, respectively. Table 1 shows the parameters acquired by the two models. Plotting log (*q*_e_*−q*_*t*_) versus *t* for uranium sorption at different temperatures yielded the *k*_1_ values. As illustrated in Fig. 9, the data from the first-order mechanism did not fit when applied to uranium biosorption by algal biomass. Figure 10 represents the kinetic curve of *t/q*_*t*_ versus *t* for uranium biosorption. The figures reveal straight lines with strong linearity and correlation coefficients closer to unity (0.985–0.99), which are greater than the pseudo-first-order correlation coefficients (0.899–0.932). As a result, the biosorption reaction can be more accurately approximated by pseudo-second-order as the dominating mechanism, meaning that the biosorption process could be chemisorption (Yuan et al. 2020; Idris et al. 2013).

**Table 1:** Kinetic parameters of uranium biosorption by *C. sorokiniana* biomass at different temperature

Temp, °C	*q*_e exp_ (mg/g)	Pseudo-first-order			Pseudo-second-order		
		*k*_1_	*q*_e cal_	*R*^2^	*k*_2_	*q*_e cal_	*R*^2^
25	185	0.0212739	178.23787	0.932	0.0003828	200.150943	0.99
35	175	0.0163916	179.01935	0.923	0.0003578	190.637464	0.99
45	165	0.0143473	179.96995	0.909	0.0003801	178.559424	0.99
55	155	0.0129384	189.88908	0.911	0.0003355	172.036319	0.985
65	145	0.0105124	185.60944	0.896	0.0003233	159.677650	0.9855

### Biosorption isotherm

**Fig. 11:**
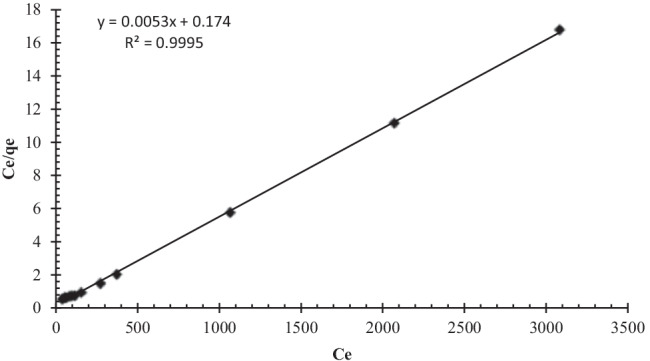
Langmuir biosorption isotherm model of uranium on algal biomass (pH: 2.5, *V*: 10 mL, *C*_0_: 450 mg/L (U), *w*: 0.09 g algal biomass, rpm: 250, contact time: 90 min)

The link between uranium ion biosorption capacity and its concentration at equilibrium was investigated using several isotherm models to correlate experimental biosorption isotherm data obtained from batch studies. In general, Langmuir and Freundlich isotherms are the most often utilized isotherm models for biosorbent use in aqueous solution. Langmuir isotherm (Langmuir 1918) explains ion biosorption to ligand sites in a single layer on the biosorbent surface with no interaction with biosorbed species. The Langmuir isotherm has the following linear form:

6 $${{c}_e}\left/{{q}_e}\right.= {1}\left/ {b.{q}_{\mathrm{max}}}\right.+{{c}_e}\left/ {{q}_{\mathrm{max}}}\right.$$

where *C*_e_ (mg/L) denotes the solute’s equilibrium concentration, *q*_e_ (mg/g) is the amount of uranium adsorbed per unit mass of the biosorbent, *q*_max_ (mg/g) denotes the maximum biosorption capacity, and *b* (L/mg) denotes the Langmuir constant. The linear plots of *C*_e_*/q*_e_ against *C*_e_ yield *q*_max_ and *b* (Fig. 11).

According to the Freundlich isotherm model, when the biosorption sites are fully occupied, the biosorption energy declines exponentially. The Freundlich isotherm represents adsorption on a heterogeneous surface as well as interactions between adsorbed molecules (multilayer biosorption) (Zhang et al. 2012).

**Fig. 12:**
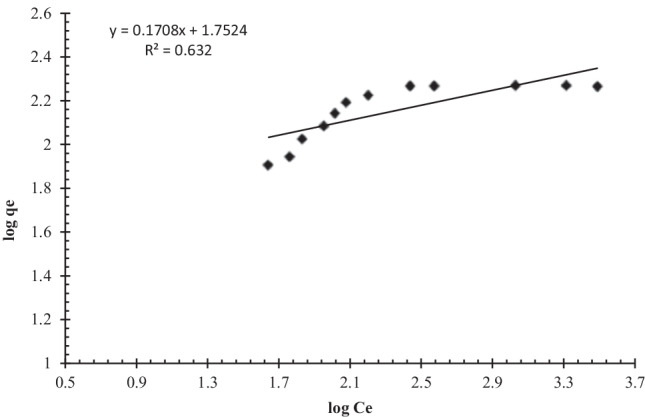
Freundlich biosorption isotherm model of uranium on algal biomass (pH: 2.5, *v*: 10 mL, *C*_0_: 450 mg/L, *w*: 0.09 g algal biomass, rpm: 250, contact time: 90 min)

Freundlich equations can be expressed in linear form as follows:

7 $$\log\ {q}_{\mathrm{e}}=\log\ {k}_f+{1}\left/{n}\right.\log {C}_{\mathrm{e}}$$

where *q*_e_ is the equilibrium biosorption capacity (mg/g), *C*_e_ is the equilibrium uranium concentration in solution (mg/L), *k*_f_ is a Freundlich constant linked to biosorption capacity, and *n* is a Freundlich constant related to biosorption intensity. The intercept and slope of the linear plot of log *q*_e_ against log *C*_e_ can be used to calculate *k*_f_ and *n* (Fig. 12).

The parameters shown in Table 2 were derived using the linear curves shown in Figures 11 and 12. The higher *R*^2^ value of Langmuir isotherm model and biosorption capacity calculated from Langmuir model (*q*_max_ = 188.7 mg/g), which was close to the value measured by the experiment (184 mg/g), both indicate that the Langmuir model can well describe the biosorption of uranium onto *C. sorokiniana* biomass (Khani 2011; Keshtkar et al. 2015). According to the isotherm study, the uranium biosorption process onto *C. sorokiniana* was most likely monolayer coverage. Another adsorption isotherm (DKR model) was used to calculate the apparent free energy and adsorption properties (Dubinin and Serpinsky 1981):

8 $$\ln {q}_{\mathrm{e}}=\ln {X}_m-{\beta \upvarepsilon}^2$$

where *q*_e_ is the adsorption capacity at equilibrium (mol g^−1^), *X*_*m*_ is the theoretical DKR monolayer adsorption capacity (mol/g), *β* (mol^2^/J^2^) is a constant associated with adsorption energy, and *ε* (J/mol) is the Polanyi potential related to the equilibrium concentration and can be defined as follows:

**Table 2:** Langmuir, Freundlich, and DKR parameters for uranium biosorption

*q*_e exp_ (mg/g)	Langmuir isotherm			Freundlich isotherm			DKR		
	*b*	*q*_e cal_	*R*^2^	*n*	*k*_f_	*R*^2^	*X*_*m*_	*E*	*R*^2^
184	0.0304	188.679	0.995	5.851	56.545	0.632	287.62	14.8	0.71

9 $$\upvarepsilon = RT\ \mathit{\ln}\left(1+{1}\left/{{C}_e}\right.\right)$$

The mean adsorption energy, *E* (kJ/mol), is the free energy change when one mole of analyte is transported from the solution to the surface of the sorbent, and it offers information regarding chemical and physical adsorption (Wang et al. 2015).

10 $$E={1}\left/ {\sqrt{2\beta }}\right.$$

**Fig. 13:**
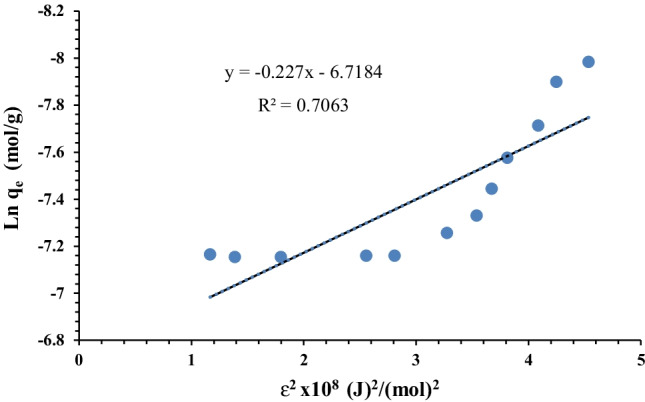
DKR biosorption isotherm model of uranium on algal biomass (pH: 2.5, *v*: 10 mL, *C*_0_: 450 mg/L, *w*: 0.09 g algal biomass, rpm: 250, contact time: 90 min)

In terms of the mean adsorption energy, *E*, adsorption is described as physisorption if it is less than 8 kJ/mol, ion exchange if it is between 8 and 16 kJ/mol and chemisorption if it is greater than 16 kJ/mol (Shen et al. 2010; Shi et al. 2015). The linear plot of the DKR model revealed that the mean free energy *E* = 14.8 kJ/mol, confirming chemisorption adsorption with ion exchange mode (Fig. 13).

Comparison of the maximum uranium biosorption capacity *q*_max_ of *C. sorokiniana* biomass with other adsorbents in the literature revealed that *C. sorokiniana* biomass was among the highest values recorded by the previous studies (Table 3).

**Table 3:** Sorption capacity from the previous work compared to our results

*q*_e_ (mg/g)	Adsorbent type	Reference
184	*Chlorella sorokiniana*	Current study
62.5	*Dictyopteris polypodioides*	Bampaiti et al. (2016)
113.5	*Saccharomyces cerevisiae*	Faghihian and Peyvandi (2012)
152	*Cladophora hutchinsiae*	Bagda et al. (2017)
97.15	*Chlorella vulgaris*	Amini et al. (2012)
190.1	*Anabaena flos-aquae*	Yuan et al. (2020)
6.789	*Aspergillus niger*	Ding et al. (2014)
29.412	*Tea waste*	Li et al. (2015)
94.30	*Ferroferric oxide/schiff base composite*	Zhang et al. (2012)
125	*Oxine functionalized magnetic Fe*_*3*_*O*_*4*_	Tan et al. (2015)
140.45	*Ceratophyllum demersum*	Yi et al. (2017a, 2017b)

### Thermodynamic parameters of biosorption

The following equations were used to derive thermodynamic parameters, which are listed in Table 4.

11 $$\ln {K}_d=\frac{\Delta \mathrm{S}}{R}-\frac{\Delta \mathrm{H}}{\mathrm{RT}}$$

**Table 4:** Thermodynamic data for biosorption of uranium ions onto *C. sorokiniana* biomass

ΔH (kJ/mol)	ΔS (kJ/mol K)	ΔG (kJ/mol)
−19.5562	−0.08552	5.92811

12 $$\Delta G=\Delta \mathrm{H}-\mathrm{T}\Delta S$$

13 $${K}_d={{q}_e}\left/{{C}_e}\right.$$

where *K*_*d*_ is the equilibrium constant and *R* is the gas constant (8.314 J mol^−1^ K^−1^) and *T* is the absolute temperature in Kelvin (K). The computed enthalpy and entropy from the plot of ln *K*_*d*_ against *1/T* were −19.5562 kJ mol^−1^ and −0.08552 J mol^−1^ K^−1^, respectively (Fig. 14). The enhanced degree of orderliness, which reflects the highest affinity of *Chlorella sorokiniana* surface for uranium, can explain negative ΔS value. The exothermic nature of the sorption process was shown by the negative ΔH value. Depending on the temperature, the Gibbs free energy, ΔG (kJ mol^−1^), could be positive or negative. The presence of a positive ΔG value indicated that the biosorption process is more favorable at lower temperatures. As a result of the increased uranium biosorption at lower temperatures, this study implies that the uranium biosorption process by algal biomass could occur spontaneously (Yi et al. 2017a, 2017b).

**Fig. 14:**
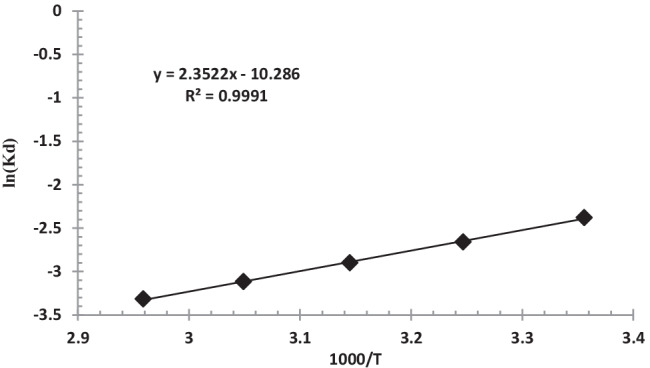
Plot of Ln *K*_d_ versus 1000/T for uranium biosorption onto *C. sorokiniana* biomass (pH: 2.5, *v*: 10 mL, *C*_0_: 450 mg/L, *w*: 0.09 g algal biomass, rpm: 250, contact time: 90 min)

### Elution of uranium

**Fig. 15:**
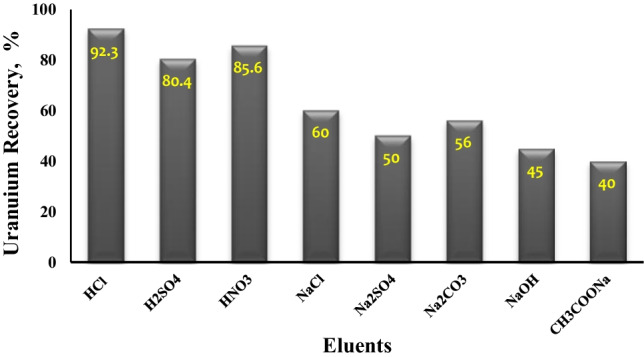
Effect of eluent type on a percentage of uranium elution

The regeneration and reuse of the biosorbent were examined by eluting uranium ions from the loaded algal biomass following the sorption process with a variety of elution solutions. To get the highest elution percentage, various solutions such as HCl, H_2_SO_4_, HNO_3_, NaCl, Na_2_SO_4_, Na_2_CO_3_, NaOH, and CH_3_COONa were utilized. At room temperature, uranium elution studies were carried out in batches. Elution studies were carried out by shaking 0.1 g of loaded algal biomass and 10 mL of 1 molar eluting reagent for 1 h/250 rpm. Figure 15 represents the graphical outcome of uranium elution from algal biomass using various solutions. According to the data obtained, it is clear that among the eluents used in this study, HCl is the most promising, one compared to H_2_SO_4_, and HNO_3_, where they give 92.3%, 80.4%, and 85.6% uranium recovery, respectively, while the others yielded low elution compared to mineral acids, indicating that biomass has a strong affinity for uranium ions. Finally, for algal biomass adsorbent regeneration, washing algal biomass with 1 molar HCl acid may be enough (Akhtar et al. 2007).

### FTIR spectra of chlorella sorokiniana biomass

The FTIR patterns of non-living biomass of *C. sorokiniana* were recorded before and after uranium bioorption to define individual functional groups involved in biosorption. The bands at approximately 3276.33 cm^−1^ corresponded to O–H and N–H stretching vibrations in *C. sorokiniana* biomass. The peaks at 1636.14 cm^−1^ and 1537.94 cm^−1^ were attributed to amino group N–H stretching. The C–OH stretching was assigned the band at 1031.52 cm^−1^ (Yi et al. 2016).

**Fig. 16:**
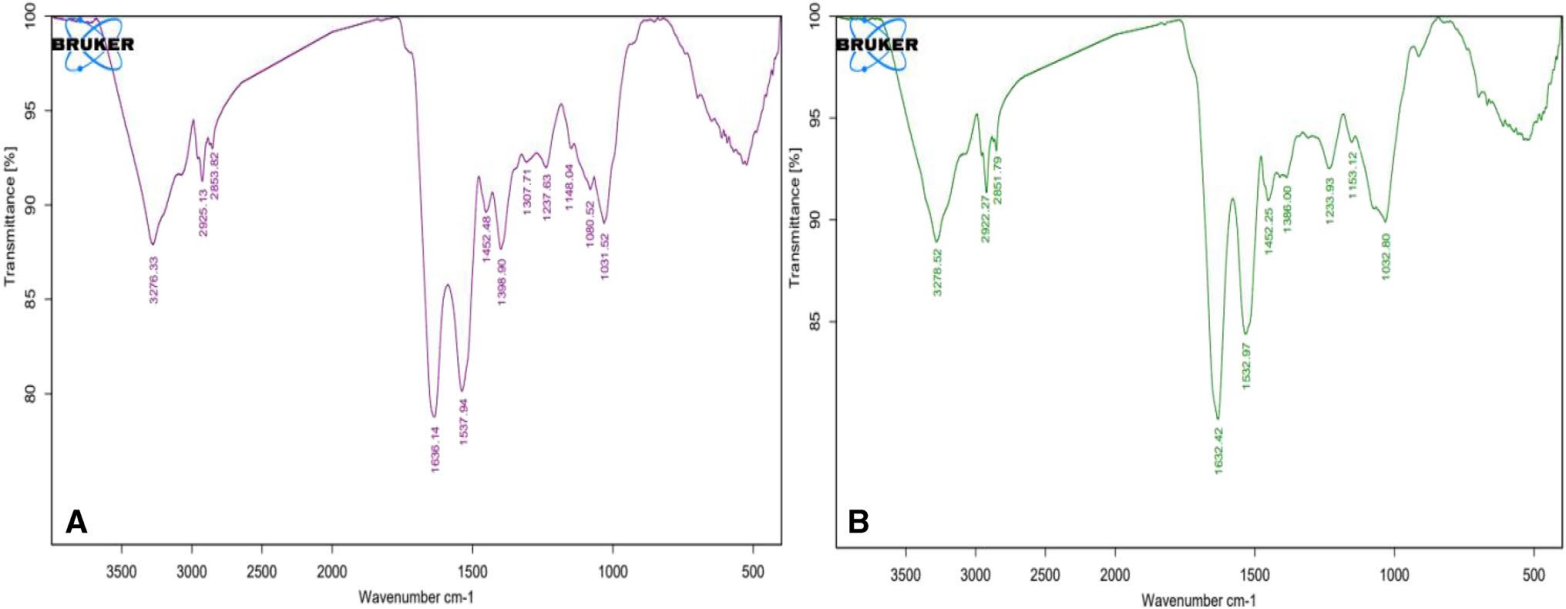
FTIR spectra of *C. sorokiniana* biomass: **A** before and **B** after uranium biosorption

Peaks at 1031.52, 1537.94, and 3276.33 cm^−1^ for uranium-loaded *C. sorokiniana* biomass correspond to –COOH, –NH2, and –OH shifted to 1032.8, 1532.97, and 3278.52 cm^−1^, respectively (Fig. 16), and there intensities were reduced. It implied that –COOH, –NH2, and –OH performed important role in uranium biosorption process.

### Energy dispersive X-ray spectroscopy

**Fig. 17:**
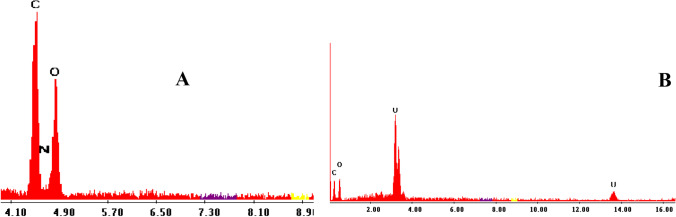
EDX spectrum of biomass **A** before uranium adsorption and **B** after biosorption of uranium

The chemical composition of the algal biomass was determined by EDX analysis before and after adsorption of uranium ions. As shown in Figure 17, the chemical composition of the adsorbent prior to adsorption consisted of C, O, and N, whereas the uranium ions were observed by EDX after the adsorption experiment, emphasizing the uptake of uranium by the algal biomass.

### Application

As previously stated, it was discovered that the optimized conditions of uranium biosorption from the sulfate synthetic solution by the prepared *C. sorokiniana* biomass had to be applied to a nuclear waste mixture. Table 5 shows the chemical composition of actual technological sample (nuclear waste mixture from the Nuclear Materials Authority) assaying 148-ppm uranium at the established optimum conditions. Using the obtained optimum parameters, we concluded that the removal biosorption efficiency was 93.4% of the calculated biosorption efficiency realized under the operating conditions. The decrease in biomass capacity following interaction with the nuclear waste mixture sample could be attributed to uranium and ion competition in the waste sample.

**Table 5:** Chemical composition of the studied nuclear waste mixture

Element	U	Ca	Fe	Mg	Zn	Ni	La
Conc. (mg/L)	148	212	53	118	15	45	86

## Conclusion

Uranium and its compounds are radioactive and toxic, as well as highly polluting and damaging the environment. Novel uranium adsorbents with high biosorption capacity that are both eco-friendly and cost-effective are continuously being researched. The non-living biomass of the fresh water green microalga *Chlorella sorokiniana* was used to study the biosorption of uranium from aqueous solution. The current study revealed that *C. sorokiniana* non-living biomass could be an efficient, rapid, low-cost, and convenient method of removing uranium from aqueous solution, since it has functional groups (carboxyl, amino, and hydroxyl) on its surface that could contribute to the uranium biosorption process, which involves ion exchange and uranium absorption, and coordination mechanisms. For algal biomass adsorbent regeneration and reuse, washing algal biomass with 1 molar HCl acid may be enough.

## Data Availability

The datasets used and/or analyzed during the current study are available from the corresponding author on reasonable request.
